# Metabolite Analysis of Jerusalem Artichoke (*Helianthus tuberosus* L.) Seedlings in Response to Polyethylene Glycol-Simulated Drought Stress

**DOI:** 10.3390/ijms22073294

**Published:** 2021-03-24

**Authors:** Mengliang Zhao, Yanjing Ren, Wei Wei, Jiaming Yang, Qiwen Zhong, Zheng Li

**Affiliations:** 1 State Key Laboratory of Crop Stress Biology for Arid Area, College of Horticulture, Northwest A&F University, Yangling 712100, China; 8304269@163.com (M.Z.); davidvvs@outlook.com (W.W.); jiamingy0112@163.com (J.Y.); 2 Academy of Agriculture and Forestry Sciences, Qinghai University, Xining 810016, China; renyan0202@163.com; 3 Qinghai Key Laboratory of Vegetable Genetics and Physiology, Xining 810016, China

**Keywords:** Jerusalem artichoke, drought stress, metabolism, metabolic network

## Abstract

Jerusalem artichokes are a perennial crop with high drought tolerance and high value as a raw material to produce biofuels, functional feed, and food. However, there are few comprehensive metabolomic studies on Jerusalem artichokes under drought conditions. Methods: Ultra-performance liquid chromatography and tandem mass spectrometry were used to identify differential metabolites in Jerusalem artichoke seedling leaves under polyethylene glycol (PEG) 6000-simulated drought stress at 0, 18, 24, and 36 h. Results: A total of 661 metabolites and 236 differential metabolites were identified at 0 vs. 18, 18 vs. 24, and 24 vs. 36 h. 146 differential metabolites and 56 common were identified and at 0 vs. 18, 24, and 36 h. Kyoto Encyclopedia of Genes and Genomes enrichment identified 236 differential metabolites involved in the biosynthesis of secondary metabolites and amino acids. Metabolites involved in glycolysis, phenolic metabolism, tricarboxylic cycle, glutamate-mediated proline biosynthesis, urea cycle, amino acid metabolism, unsaturated fatty acid biosynthesis, and the met salvage pathway responded to drought stress. Conclusion: A metabolic network in the leaves of Jerusalem artichokes under drought stress is proposed. These results will improve understanding of the metabolite response to drought stress in Jerusalem artichokes and develop a foundation for breeding drought-resistant varieties.

## 1. Introduction

Drought stress is a major direct environmental factor, adversely affecting agricultural crop productivity and economic losses worldwide, with the effects of drought ranking first among all natural disasters [1,2,3,4]. As a result of the increasing world population, urban expansion, and the scarcity of global water, an increasing amount of arable land has lost the ability to produce food and forced agricultural production into marginal areas such as desert and land subjected to soil salinization [5,6]. Therefore, enhancing drought resistance in agricultural crop breeding is one of the most important goals.

Jerusalem artichoke (*H. tuberosus* L.) is a hardy perennial crop in the family Asteraceae, with high adaptability to barren lands, such as salinized land and soil with high sediment, and is suitable for growing in dry, cold, and sunny climates [7,8]. Jerusalem artichokes are an important cash crop and can be used as human food [9], animal feedstock [10,11], and raw materials in the production of pharmaceuticals, cosmetics, biofuels [12], and ecological restoration [13]. Because of its diverse uses and high adaption to infertile lands, Jerusalem artichokes have become a rapidly developing agricultural crop in recent years.

Although the statistics are incomplete, the total number of metabolites in plants is approximately 200,000 [14,15]. Changes in the variety and number of metabolites can reflect the adaptability of plants to the environment [16,17]. When faced with drought stress, plants usually respond by leaf rolling, stomatal closure, and inhibition of growth and development [18], meanwhile, some metabolites closely related to these responses have changed [19,20]. In rice, Ma et al. [21] found that metabolites play vital roles in protecting photosynthesis under dehydration by osmotic adjustments and/or antioxidant mechanisms, and considered 4-hydroxycinnamic acid and ferulic acid as key metabolites for drought tolerance. Chmielewska et al. [22] found that the highest number of drought-responsive compounds were amino acids under drought-induced metabolomic changes in barley. Ullah et al. [23] found that sugars, amino acids, organic acids, and low molecular weight compounds increased in leaf and root samples under drought stress in Triticeae species. Goufo et al. [24] considered that proline, galactinol, and quercetin derivatives responded the most to drought, and they found that these metabolites accumulated similarly in the leaves of cowpea, but differently in roots, suggesting a more conservative strategy to cope with drought in aerial parts.

Pan et al. [25] found the differential expression of lipids, amino acids, organic acids, carbohydrates, and carbohydrate conjugates; nucleotides and their analogs; indoles and their derivatives; alkaloids and their derivatives; amine compounds, pyridines, and their derivatives in the metabolic processes of two annual ryegrass under drought stress. Gomes et al. [26] discovered that a total of 14 metabolites increased exclusively in cowpea, including sucrose, fucose, urea, alanine, and putrescine. Metabolomics are an effective method to explain plant growth and adaptation mechanisms in harsh environments [27,28], and has been widely used to investigate plant response to abiotic stresses. To the best of our knowledge, few studies have reported the metabolite changes that respond to drought stress in Jerusalem artichokes. Herein, Jerusalem artichoke seedling leaves under polyethylene glycol (PEG) 6000-simulated drought stress were used to perform a comprehensive analysis of metabolite changes by widely targeted technology with ultra-performance liquid chromatography and tandem mass spectrometry (UPLC-MS/MS). The results reported here will provide an improved understanding of the metabolite response to drought stress in Jerusalem artichokes and develop a foundation for breeding drought-resistance in Jerusalem artichoke varieties.

## 2. Results

### 2.1. Physiological Changes of Jerusalem Artichoke in Response to Drought Stress

**Figure 1:**
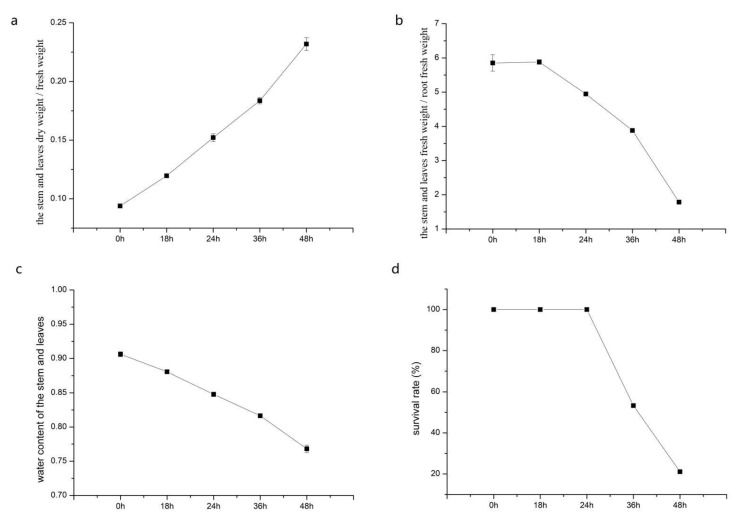
Physiological changes of Jerusalem artichoke in response to drought stress. (**a**) Changes of the stem and leaves dry weight/fresh weight from 0 to 48 h; (**b**) changes of the stem and leaves fresh weight/root fresh weight from 0 to 48 h; (**c**) changes of the water content of stems and leaves from 0 to 48 h; (**d**) changes of the survival rate in the Jerusalem artichoke from 0 to 48 h.

**Figure 2:**
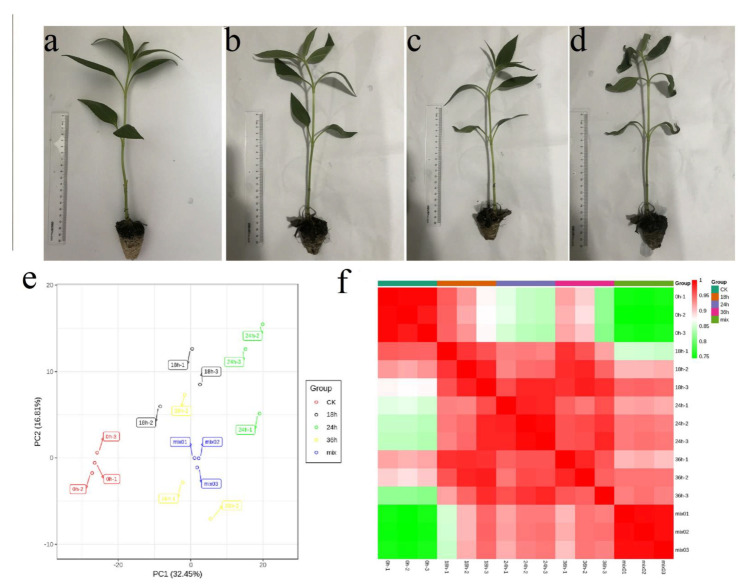
The morphology of Jerusalem Artichokes treated with drought stress, and the heatmap of correlation and principal component analysis (PCA) between different drought treatment and quality control (QC) samples. (**a**) 0 h; (**b**) 18 h; (**c**) 24 h; (**d**) 36 h; (**e**) Principal component analysis. (**f**) Heatmap of correlation.

To select the appropriate stress time, we obtained the stem and leaves dry weight/fresh weight, the stem and leaves fresh weight/root fresh weight, and the water content of stems and leaves in Jerusalem artichokes under PEG 6000-simulated drought stress at 0, 18, 24, 36, and 48 h (Figure 1a–c). With the treatment time increased, the stem and leaves dry weight/fresh weight showed an upward trend from 0.094 ± 0.002 to 0.232 ± 0.005. On the contrary, the stem and leaves fresh weight/root fresh weight and water content of stems and leaves showed a downward trend as the treatment time increased. At the same time, Jerusalem artichoke seedlings under drought stress at 18, 24, 36, and 48 h were rehydrated and the survival rate was counted (Figure 1d). The results showed that the survival rate was 100% from 0–24 h, while it reduced to 53.3% at 36 h and 21.1% at 48 h. Based on the physiological data, three stress treatment times (0, 18, 24, and 36 h) were selected, and the physiological changes are shown in Figure 2a–d.

### 2.2. Qualitative and Quantitative Metabolites

To ensure the repeatability of samples under drought stress at different times, quality control (QC) samples were inserted in every 10 test samples during the analysis [29]. The overlapping map of total ion current (TIC) and different QC samples showed that the curve overlap of total ion flow was high and the retention time and peak intensity were the same, which indicates that the signal stability for the same sample was better when the mass spectrometry was detected at different times (Figure S1).

The qualitative and quantitative mass spectrometry analysis of metabolites in different drought stress samples were based on the Kyoto Encyclopedia of Genes and Genomes (KEGG) compound database, MetWare database (MWDB), and multiple reaction monitoring (MRM). A total of 661 metabolites were obtained using widely targeted metabolic analysis (Table S1). The classification of secondary substances showed that there were 28 groups, including 127 phenolic acids, 77 amino acids and derivatives, 60 organic acids, 45 nucleotides and derivatives, 41 free-fatty acids, 41 flavonoid, 37 saccharides and alcohols, 36 flavonols, 28 glycerol esters, 21 lysophosphatidylcholine, 14 isoflavones, 13 vitamins, 12 lysophosphatidylethanolamine, 12 sesquiterpenoids, nine triterpene, seven alkaloids, six coumarins, six tannins, four lignans, three plumeranes, three anthocyanins, three phosphatidylcholines, two flavonoid carbonosides, one dihydroflavonols, one phenolamine, one flavanol, one sphingolipid, and 50 other metabolites (Table S2).

### 2.3. PCA and PCC Analysis

Principal component analysis (PCA) was carried out on the samples (including QC samples) to understand the overall metabolic differences between the samples and the variation in the group. PCA measures a separation in the trend of metabolic groups between groups, suggesting whether there are differences between metabolic groups or not [30]. The PCA of different drought stress treatments and QC samples showed that there was little variation between each drought stress treatment sample, and revealed that a trend of separation was evident between drought stress treatment at different times. Those data suggested that the metabolic differences between drought stress treatments at different times were significant (Figure 2e). Pairwise analysis of metabolic differences between drought treatments was also performed. The results showed that the metabolism between drought treatments at different times was clearly separated in the first component (PC1), thus, indicating drought stress treatment significantly affected the metabolism of *H. tuberosus* (Figure S2).

A heatmap of the correlation between the three biological replicates and QC samples was also constructed, and the Pearson correlation coefficient (PCC) R was used as an evaluation index of biological repetition correlation. The data all revealed a highly significant positive correlation between the three biological replicates (Figure 2f).

### 2.4. Orthogonal Partial Least Squares-Discriminant Analysis

**Figure 3:**
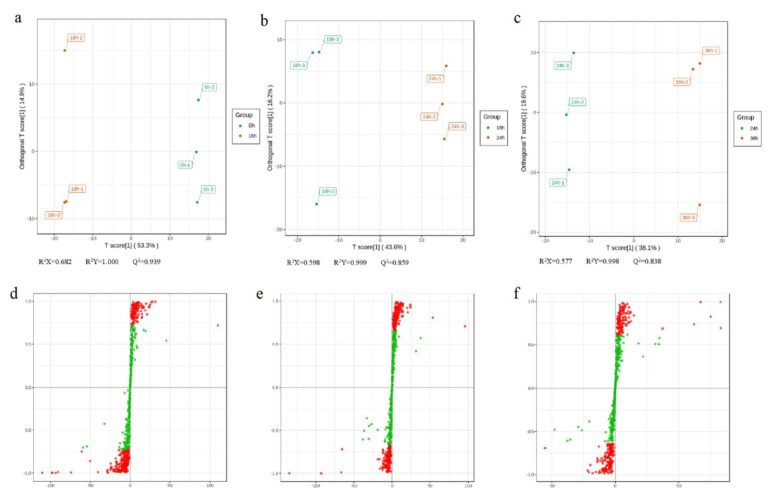
Orthogonal partial least squares-discriminant analysis (OPLS–DA) scores. Scores of the OPLS–DA model with (**a**) 0 vs. 18 h, (**b**) 18 vs. 24 h, (**c**) 24 vs. 36 h. OPLS–DA S-plot model with (**d**) 0 vs. 18 h, (**e**) 18 vs. 24 h, (**f**) 24 vs. 36 h. R^2^ Y scores and Q^2^ values represent the interpretation rate of the model to the Y matrix and the prediction ability of the model, respectively. When Q^2^ > 0.5, the model can be considered an effective model, and Q^2^ > 0.9 is an excellent model.

To search for differential metabolites, orthogonal partial least squares-discriminant analysis (OPLS-DA) was used to extract the components in the Y of independent variable X and dependent variable, and then calculate the correlation between components. OPLS-DA combines orthogonal signal correction (OSC) and PLS-DA methods, which decompose X matrix information into two types related to Y and irrelevant differences [31]. Then differential variables are screened by removing the irrelevant differences. Results showed that R^2^X were all higher than 0.55, R^2^Y scores were all higher than 0.99, and Q^2^ values were all larger than 0.83 in the 0 vs. 18, 18 vs. 24, and 24 vs. 36 h samples, respectively (Figure 3a–c), confirming that the differential metabolites responded to drought stress treatment. The S-plot map of OPLS-DA is shown in Figure 3d–f. The OPLS-DA model was also verified using 200 random permutations and combination alignment tests.

### 2.5. Differential Metabolites Analysis and Enrichment

**Figure 4:**
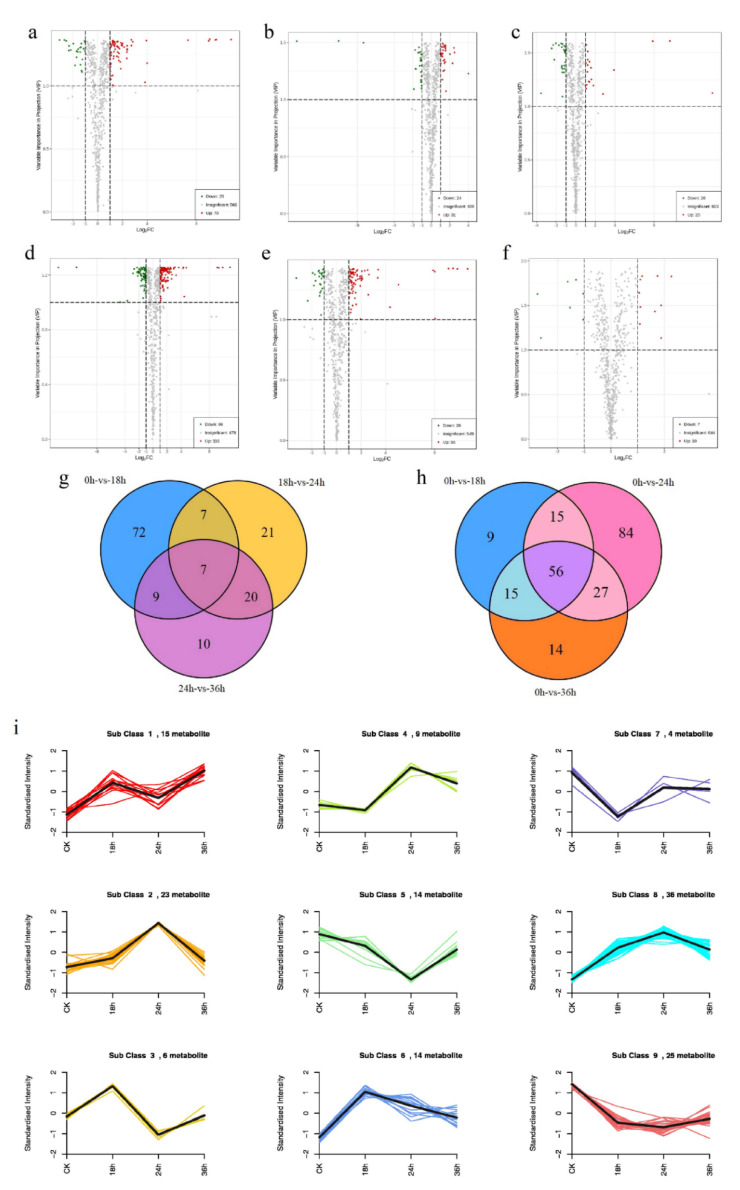
Differential metabolite analysis of Jerusalem artichokes under drought conditions. Volcano maps of differential metabolites in different pairwise comparisons: (**a**) 0 vs. 18 h; (**b**) 18 vs. 24 h; (**c**) 24 vs. 36 h; (**d**) 0 vs. 24 h; (**e**) 0 vs. 36 h; (**f**) 18 vs. 36 h. (**g**) Venn diagram of differential metabolites in a multiple pairwise comparison of 0 vs. 18 h, 18 vs. 24 h, and 24 vs. 36 h. (**h**) Venn diagram of differential metabolites in multiple pairwise comparisons of 0 vs. 18 h, 0 vs. 24 h and 0 vs. 36 h. (**i**) Trend analysis of differential metabolites during the treatment of drought stress from 0 to 36 h.

Differential metabolites were screened by combining variable importance in projection (VIP) and fold change in all six pairwise comparisons, and 236/661 differential metabolites were detected (Table S3). Results showed that there were 95 differential metabolites (70 up- and 25 down-regulated) at 0 vs. 18 h, 55 differential metabolites (31 up- and 24 down-regulated) at 18 vs. 24 h, 46 differential metabolites (20 up- and 26 down-regulated) at 24 vs. 36 h, 182 differential metabolites (116 up- and 66 down-regulated) at 0 vs. 24 h, 112 differential metabolites (86 up- and 26 down-regulated) at 0 vs. 36 h, and 27 differential metabolites (10 up- and 17 down-regulated) at 18 vs. 36 h. Volcano maps of differential metabolites in different pairwise comparisons are shown in Figure 4a–f. According to the log_2_fold change of metabolites in different pairwise comparisons, the results of differential metabolites that rank changes ahead are shown in Figure S3.

After searching the differential metabolites in all pairwise comparisons, the common differential metabolites in 0 vs. 18, 18 vs. 24, and 24 vs. 36 h were then searched. Seven common differential metabolites were identified and shown in the Venn diagram (Figure 4g, Table S4). A total of 146 differential metabolites were identified in all three comparisons in 0 vs. 18, 18 vs. 24, and 24 vs. 36 h. These metabolites were divided into 11 groups: amino acid and their derivatives, phenolic acids, lipids, organic acids, nucleotides and their derivates, lignans and coumarins, flavonoids, alkaloids, terpenoids, tannins, and others (Table 1). Meanwhile, the common differential metabolites in 0 vs. 18, 24, and 36 h were also searched. Fifty-six common differential metabolites were identified and are shown in the Venn diagram (Figure 4h, Table S5). A total of 221 differential metabolites were identified in all three comparisons in 0 vs. 18 h, 24, and 36 h. These metabolites were divided into 11 groups, including amino acids and their derivatives, phenolic acids, lipids, organic acids, nucleotides and their derivates, lignans and coumarins, flavonoids, alkaloids, terpenoids, tannins, and others (Table 1).

To identify the trends associated with differential metabolites and drought stress at different times, group trend analysis was performed. The results revealed that the trends could be divided into nine sub-classes. A total of 15, 23, 6, 9, 14, 14, 4, 36, and 25 metabolites were clustered from Sub-Classes 1 to 9, respectively (Figure 4i). In Sub-Class 2 and 8, the changes in metabolites increased from 0 to 24 h and decreased from 24 to 36 h. In Sub-Class 5, the changes in metabolites decreased from 0 to 24 h and increased from 24 to 36 h. In Sub-Class 6, changes in metabolites increased from 0 to 18 h and decreased from 18 to 36 h.

According to the KEGG pathway database, differential metabolites were enriched in different pathways. Differential metabolites in different pairwise comparisons were involved in metabolic pathways, biosynthesis of secondary metabolites, biosynthesis of amino acids, and ATP binding cassette (ABC) transporters (Figure S4).

### 2.6. Drought-Induced Metabolic Pathway of Amino Acids and Derivatives

Amino acids and derivatives were identified as one of the most differential metabolites in Jerusalem artichoke seedling leaves under PEG-simulated drought stress. In addition to N-acetyl-L-glutamic acid, N-acetyl-L-tryptophan, and L-ornithine, other amino acids and derivatives (L-serine, L-valine, L-threonine, L-phenylalanine, L-asparagine, L-glutamine, L-lysine, S-allyl-L-cysteine, alanylleucine, L-phenylalanyl-L-phenylalanine, N-acetyl-L-leucine, L-arginine, homoarginine, L-glutaminyl-L-valyl-L-valyl-L-cysteine, N6-acetyl-L-lysine, cyclo (Tyr-Ala), 2,6-diaminopimelic acid and oxidized glutathione) were all up-regulated under drought stress. Two prolines, trans-4-hydroxy-L-proline and glycyl-L-proline were insignificantly accumulated under 18 h of drought stress, but significantly accumulated under 24 h of drought stress.

### 2.7. Drought-Induced Accumulation of Secondary Metabolites

Phenolic acids are a type of secondary metabolite containing phenolic rings. In this study, we identified 18 phenolic acids compounds with significant drought-induced accumulation in the leaves of Jerusalem artichoke, including 4-hydroxyacetophenone, cinnamic acid, 2-methoxybenzoic acid, 3,4-dihydroxyacetophenone, caffeic acid, caffeoylmalic acid, 3-O-caffeoylshikimic acid, 4-hydroxy-3,5-diisopropylbenzaldehyde, sinapoyl malate, glucosyringic acid, sinapic acid-glycoside, 3,3′-di-O-methyl ellagic acid-4′-O-arabinoside, vanillin, syringic aldehyde, 3-O-p-coumaroylshikimic acid, 3-hydroxy-4-isopropylbenzylalcohol 3-glucoside, dicaffeoylquinic acid-glucoside, and 3,3′-di-O-methyl ellagic acid-4′-O-arabinoside. Most of these metabolites are involved in the phenolic acid branch of phenylpropanoid metabolism and further involved in flavonoid biosynthesis. Three flavonoid compounds, chrysoeriol-6,8-di-C-glucoside, quercetin-3-O-(6″-O-malonyl)-galactoside, and 6-hydroxykaempferol-7,6-O-diglucoside, were found to accumulate significantly in the drought-tolerant leaves of Jerusalem artichokes. The significant accumulation of these flavonoid compounds in the leaves of Jerusalem artichokes may act as endogenous antioxidants in the defense mechanisms of plants under drought stress.

### 2.8. Analysis of Comprehensive Metabolic Networks under Drought Stress

**Figure 5:**
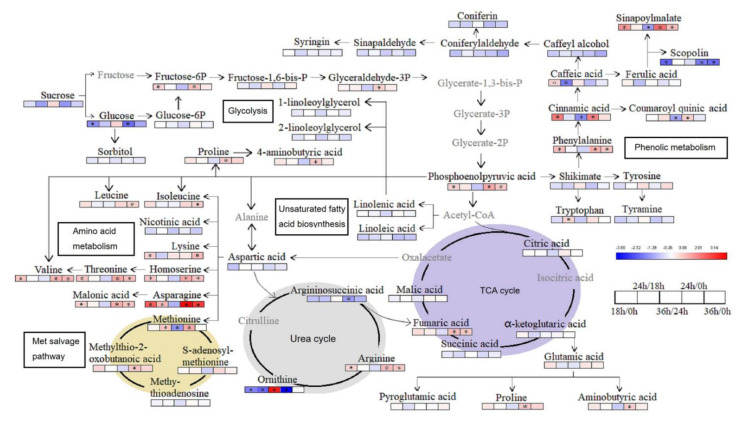
Analysis of metabolic networks in the leaves of Jerusalem artichoke under drought stress. Proposed metabolic pathways were based on the literature and web-based database of metabolic pathways. The metabolites written in gray were not detected in this study. The differential metabolite changes were represented by the log_2_ ratio. Blue represents a decrease in content and red represents an increase in content. * indicates a significant difference.

To achieve a comprehensive understanding of metabolite changes under PEG-simulated drought stress, we proposed a metabolic pathway based on the literature and web-based database of metabolic pathways. The major known pathways include glycolysis, phenolic metabolism, the tricarboxylic (TCA) cycle, glutamate-mediated proline biosynthesis, the urea cycle, amino acid metabolism, unsaturated fatty acid biosynthesis, and the met salvage pathway (Figure 5).

We identified nine metabolites related to the glycolysis pathway, including sucrose, glucose, glucose-6-phosphate, glucose-1,6-bisphosphate, fructose-6-phosphate, fructose-1,6-bisphosphate, glyceraldehyde-3-bisphosphate, phosphoenolpyruvic acid, and sorbitol. Compared with 0 h treatment, fructose-6-phosphate significantly increased at 18 and 24 h, glyceraldehyde-3-bisphosphate significantly increased at 24 h, and phosphoenolpyruvic acid significantly increased at 18, 24, and 36 h. In contrast, glucose significantly decreased at 18 and 24 h under drought stress. Phosphoenolpyruvic acid converted to acetyl coenzyme A and shikimate separately. Acetyl coenzyme A entered the TCA cycle. Metabolites involved in the TCA cycle were citric acid, α-ketoglutaric acid, succinic acid, fumaric acid, and malic acid, of which fumaric acid revealed a significant increase under drought stress. Proline and aminobutyric acid are biosynthesized by the glutamate-mediated pathway from α-ketoglutaric acid, which is increased significantly at 24 h under drought stress rather than 18 h, suggesting that 24 h treatment can effectively stimulate the proline response to PEG-simulated drought stress in Jerusalem artichokes. Shikimate can convert to phenylalanine and enter phenolic metabolism. Phenylalanine content increased significantly at 18, 24, and 36 h under PEG-simulated drought stress. Similarly, cinnamic acid content increased significantly at 18 and 24 h, caffeic acid content significantly increased at 18 h, and commaroylquinic acid content significantly increased at 24 h. Sinapoylmalate was also detected in this study and increased significantly at 18, 24, and 36 h under PEG-simulated drought stress. In the urea cycle, arginine content significantly increased at 18, 24, and 36 h under PEG-simulated drought stress, while ornithine content decreased significantly. Additionally, met salvage pathway metabolites were also found to be involved in the response to PEG-simulated drought stress, of which methylthio-2-oxobutanoic acid and s-adenosyl methionine increased at 18, 24, and 36 h.

## 3. Discussion

Drought stress is one of the most important stress factors affecting plant growth. When confronted with drought stress, plants have evolved a variety of mechanisms to respond [22]. It is well known that amino acids are an important nitrogen source for plants. Many researchers have reported that specific amino acids may be able to defer protein degradation under drought stress [32,33,34]. The significant accumulation of amino acids and derivatives regulates plant defense against drought stress by osmotic balancing and maintaining the stability of the cell membrane structure [35,36]. Jia et al. [37] identified 16 amino acids and their derivatives, and the concentration of most amino acids (valine, threonine, isoleucine, glycine, arginine, alanine, asparagine, leucine, and tyrosine) increased in response to the water deficit within 6 to 10 days. Following this, concentrations then reached an approximate plateau, and decreased to the control level on the 14th day. They also found that most of the amino acids were significantly enriched under moderate stress, but dramatically decreased under severe stress. Ullah et al. [23] found that amino acids and derivatives increased in both leaf and root samples of Triticeae species under drought stress. Yang et al. [38] found that 132/141 amino acid metabolites detected were significantly altered by drought stress treatment in developing maize kernels. Law [39] summarized that amino acids were the common response to drought observed in poplar. All these proved that amino acids and their derivatives were the main factors responding to drought stress in plants.

Proline, an osmotic regulator substance, is supposed to enhance cell tolerance and defend cell damage from various abiotic stresses [40,41]. Chmielewska et al. [22] analyzed the changes in the metabolites of barley under drought stress and identified the accumulation of proline in leaves of both tested barley cultivars subjected to drought. Moschen et al. [42] identified the metabolites involved in drought stress response in sunflower and found that proline showed higher levels under drought conditions. Wang et al. [4] reported that proline was accumulated significantly in common wild soybean, and especially in drought-tolerant wild soybean (*p* < 0.01). Koobaza et al. [43] detected changes in metabolites in desiccation tolerance in the young seedlings of wheat and revealed that the stress increased the proline content of the seedlings, which was twice as high in the T2 (20 d) than in the T1 (10 d) seedlings. The high content of proline in plants subjected to drought stress implied that proline has a vital role in response to drought stress.

Phenylalanine, a precursor of many key secondary metabolite pathways, such as flavonoids and anthocyanins, affects cell osmotic regulation and improves plant drought tolerance [44,45,46]. The accumulation of phenylalanine was significant in the leaves of Jerusalem artichokes induced by drought stress in this report. Wang et al. [4] reported that aromatic amino acid phenylalanine accumulated significantly in drought-tolerant wild soybean. Jia et al. [34] identified the significant drought-induced accumulation of phenylalanine in poplar plants. The high levels of phenylalanine induced by drought suggest that this amino acid is important for the adaptation of Jerusalem artichokes to drought stress. Flavonoids, a major secondary metabolite in plants, have various functions in plant development and in response to biotic and abiotic stress [47,48]. Nalabayashi et al. [49] demonstrated that the accumulation of flavonoids was a key factor to enhance tolerance to oxidative and drought stresses in *Arabidopsis*. Swarcewicz et al. [50] identified that a substantial increase in the concentration of the metabolite cinnamic acid was observed in the leaves and roots of barley under drought stress. We also detected that cinnamic acid and caffeic acid significantly increased under PEG-simulated drought stress, indicating the key role of flavonoids in response to drought stresses.

In this study, a comprehensive metabolite network was proposed based on the literature and web-based database. However, changes in metabolites cannot explain the exact mechanism involved in the Jerusalem artichoke response to drought stress. More work is needed to characterize the biological function of the proposed network, such as the identification of metabolite function and protein changes. Through these, we will further reveal the molecular mechanisms of Jerusalem artichoke in response to drought stress.

## 4. Materials and methods

### 4.1. Sample Preparation

The Jerusalem artichoke cultivated variety, Qingyu No. 2, provided by the Research and Development Center of Jerusalem Artichokes, the Academy of Agriculture and Forestry Sciences of Qinghai University, were planted in a plate in the field of the Academy of Agriculture and Forestry Sciences of Qinghai University, Xining, China, in 2020. The seedlings plates were moved into a light incubator (Thermo Fisher Scientific, Waltham, MA, USA) to be cultured under light when the seedlings grew six leaves. The temperature was 25 °C during the day and 16 °C at night in the light incubator for three days. On the third day, we watered the seedlings until the soil was saturated and did not contain much water. Then the temperature was changed to 25 °C all day and the light intensity was 8000 Lux. Twenty hours later, samples of the control leaves (0 h) were collected and all the seedlings were watered with 25% PEG 6000. Then, samples were collected at 18, 24, 36, and 48 h after PEG 6000-simulated drought stress.

### 4.2. Phenotypic Data Determination

The stem, leaves, and root wet weight of Jerusalem artichokes under PEG 6000-simulated drought stress at 0, 18, 24, 36. and 48 h were weighed using an electronic balance (1/1000). The stem and leaves were then dried to a constant weight and weighed with an electronic balance (1/1000). Each set of data included five individual plants. The stem and leaves wet weight/root wet weight, the stem and leaves wet weight/dry weight and water content of the stem and leaves were measured. At the same time, Jerusalem artichoke seedlings under drought stress for 18, 24, 36, and 48 h were rehydrated and the survival rate was determined. Each set contained three replications and each replication contained 30 individual plants.

### 4.3. Appropriate Stress Time and Sample Collection

Based on the phenotypic data of Jerusalem artichokes under PEG 6000-simulated drought stress, we chose the appropriate stress time and collected Jerusalem artichoke leaves. Each biological replicate contained nine individual plants under drought stress, and the collected leaves were immediately frozen and stored at −80 °C. Sample leaves were from the third to fourth leaves from top to bottom.

### 4.4. Sample Extraction and UPLC Conditions

Sample extraction was performed following the method by Li and Song [29]. The sample extracts were then analyzed using a UPLC-ESI-MS/MS system (UPLC, Shim-pack UFLC Shimadzu CBM30A system; MS, Applied Biosystems 4500 Q TRAP) with the same protocol [29]. The effluent was alternatively connected to an ESI-triple quadrupole-linear ion trap (QTRAP)-MS.

### 4.5. ESI-Q TRAP-MS/MS Analysis Conditions

LIT and triple quadrupole (QQQ) scans were acquired on a triple quadrupole-linear ion trap mass spectrometer (Q TRAP), API 4500 Q TRAP UPLC/MS/MS system. The ion spray voltage was 5500 V in the positive ion mode and -4500 V in the negative ion mode, the source temperature was 550 °C, the ion source gas I (GSI), gas II (GSII), and curtain gas (CUR) were set at 50, 60, and 30.0 psi, respectively, and the collision gas (CAD) was high. In the QQQ, collision gas (nitrogen) was set to 5 psi.

### 4.6. Qualitative and Quantitative Analysis of Metabolites

Metabolites were qualitatively and quantitatively analyzed by mass spectrometry using a public metabolite database and the self-built MWDB. The detected metabolites were shown in the multi-reaction monitoring mode MRM metabolite detection multi-peak map. Each mass spectrum peak of different colors represents a detected metabolite. The characteristic ions of each metabolite were obtained by triple four-stage rod screening, the signal intensity of the characteristic ions was obtained in the detector, and MultiQuant software was used to integrate and correct the chromatographic peaks. The peak area (area) of each chromatographic peak represents the relative content of the corresponding metabolite [51].

### 4.7. Data Quality Assessment

To detect the repeatability of samples under drought stress, QC samples (mixed sample extracts) were inserted in every 10 test samples during the analysis [29]. The accuracy and reproducibility of metabolite detection could be determined by using the overlapping display analysis of mass spectrometry TIC of different QC samples.

### 4.8. PCA, PCC, and OPLS-DA Analysis

PCA was performed by the statistics function of prcomp within R (www.r-project.org, 8 March 2021). The PCC between samples was calculated by the cor function in R and presented as heatmaps. Orthogonal partial least squares-discriminant analysis (OPLS-DA) was used for the initial screening of differential metabolites. The OPLS-DA was calculated by the MetaboAnalystR package in R software. The prediction parameters of the OPLS-DA evaluation model are R^2^X, R^2^Y, and Q^2^, where R^2^X and R^2^Y represent the interpretation rate of the model to the X and Y matrix respectively, and Q^2^ indicates the prediction ability of the model.

### 4.9. Differential Metabolite Analysis

Differential metabolites were screened by combining the VIP in the OPLS-DA results and *p*-value or differential multiple value (fold change). VIP values containing score plots and permutation plots were generated using the R package, MetaboAnalystR. The data were log transformed (log2) and mean centering was performed before OPLS-DA. To avoid overfitting, a permutation test (200 permutations) was performed. Significantly regulated metabolites between groups were determined by VIP ≥1 and fold changes ≥2 or ≤0.5. Selected differential metabolites were further annotated in the KEGG compound database (http://www.kegg.jp/kegg/compound/, 28 November 2020). Annotated metabolites were then mapped to the KEGG pathway database (http://www.kegg.jp/kegg/pathway.html, 28 November 2020) [52].

## 5. Conclusions

Our study revealed differential metabolites in response to the PEG-simulated drought stress in Jerusalem artichokes. A total of 236/661 metabolites were identified. KEGG enrichment showed that these differential metabolites were involved in the biosynthesis of secondary metabolites and biosynthesis of amino acids. Comprehensive understanding of metabolite changes under PEG-simulated drought stress showed that the glycolysis, phenolic metabolism, tricarboxylic (TCA) cycle, glutamate-mediated proline biosynthesis, urea cycle, amino acid metabolism, unsaturated fatty acid biosynthesis, and the met salvage pathway were involved in the response to drought stress, and a metabolic network in the leaves of Jerusalem artichokes under drought stress was proposed. This study will provide an improved understanding of the metabolite response to drought stress in Jerusalem artichokes, and develop a foundation for breeding drought-resistant Jerusalem artichoke varieties.

## Figures and Tables

**Table 1:** Number of differential metabolites in the leaves of Jerusalem artichoke with drought stress.

Group Class	0 vs. 18 h		18 vs. 24 h		24 vs. 36 h		0 vs. 24 h		0 vs. 36 h	
	Up	Down	Up	Down	Up	Down	Up	Down	Up	Down
Amino acid and its derivatives	18	2	10	0	3	5	28	3	27	1
Phenolic acids	12	4	3	10	8	5	14	20	11	4
Organic acids	6	2	3	2	2	2	20	6	12	3
lipids	10	5	5	2	3	5	22	7	13	10
Nucleotide and its derivates	5	3	3	2	0	3	10	2	5	2
Lignans and coumarins	0	0	3	2	0	2	2	1	0	0
Flavonoids	1	2	0	1	0	1	3	9	2	2
Alkaloids	3	0	2	1	1	1	3	2	3	0
Tannins	2	0	0	0	0	0	3	0	2	0
Terpenoids	1	0	1	1	0	1	2	1	0	1
Others	12	7	1	4	3	1	10	15	10	3
Total	95		55		46		182		112	

## Supplementary Materials

The following are available online at https://www.mdpi.com/1422-0067/22/7/3294/s1.
