# Supplementary figures and images for: Metabolite Analysis of Jerusalem Artichoke (Helianthus tuberosus L.) Seedlings in Response to Polyethylene Glycol-Simulated Drought Stress
Source: Int J Mol Sci. 2021 Mar 24;22(7):3294. doi: 10.3390/ijms22073294 (PMC8037225; doi:10.3390/ijms22073294)

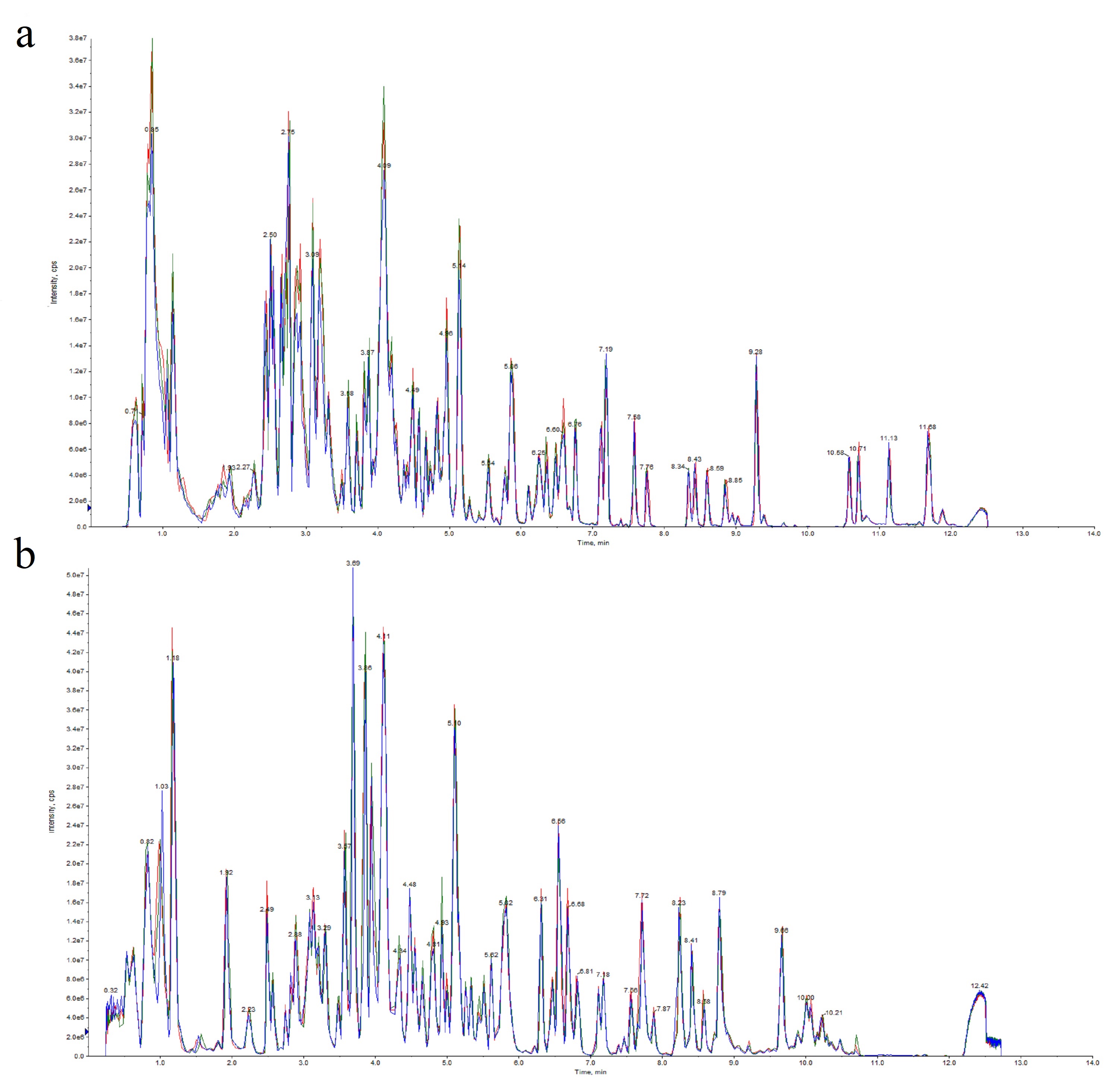

Supplement: Supplementary file 1 [file ijms-22-03294-s001.zip › supplementary files/Figure S1.jpg]

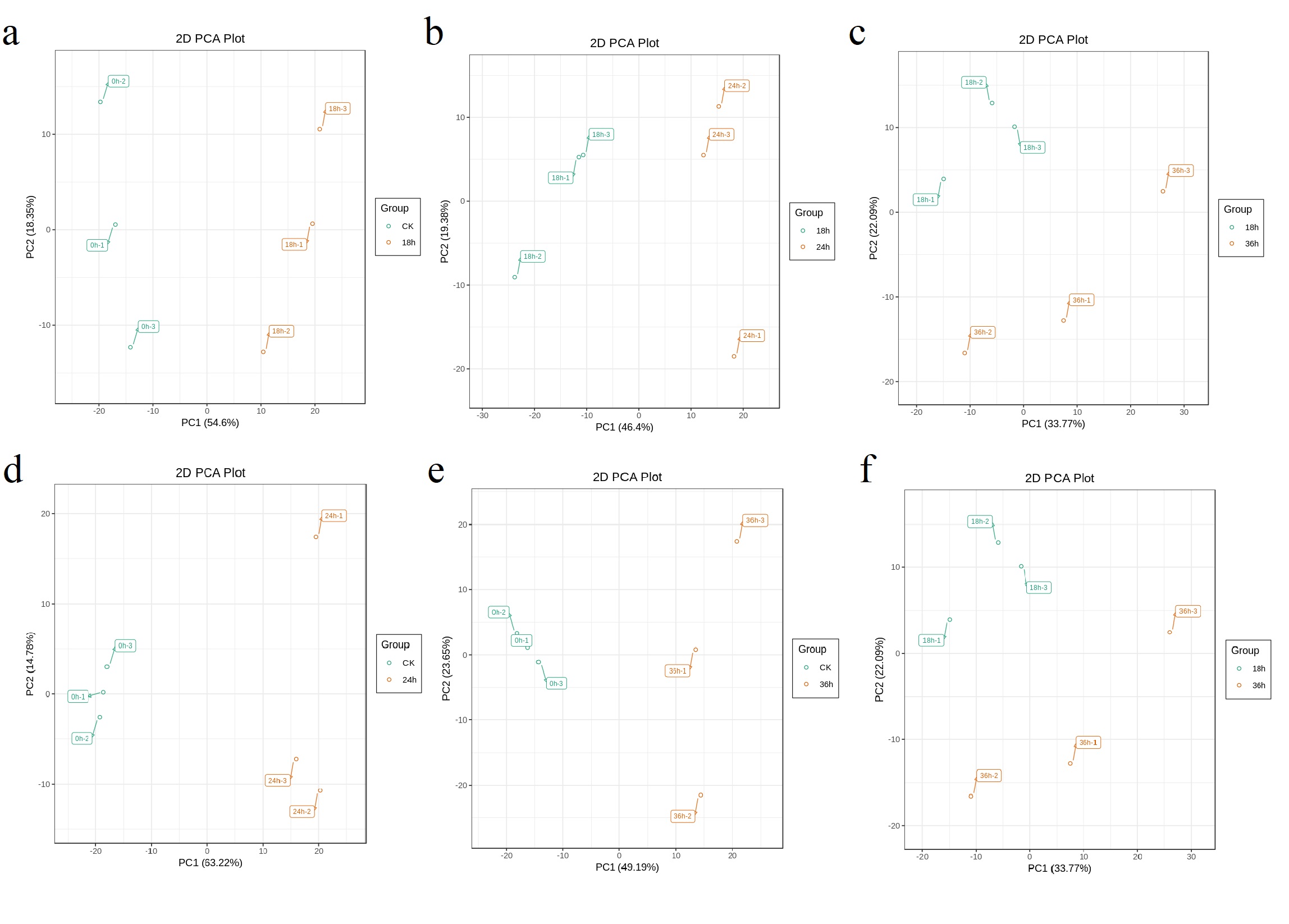

Supplement: Supplementary file 1 [file ijms-22-03294-s001.zip › supplementary files/Figure S2.jpg]

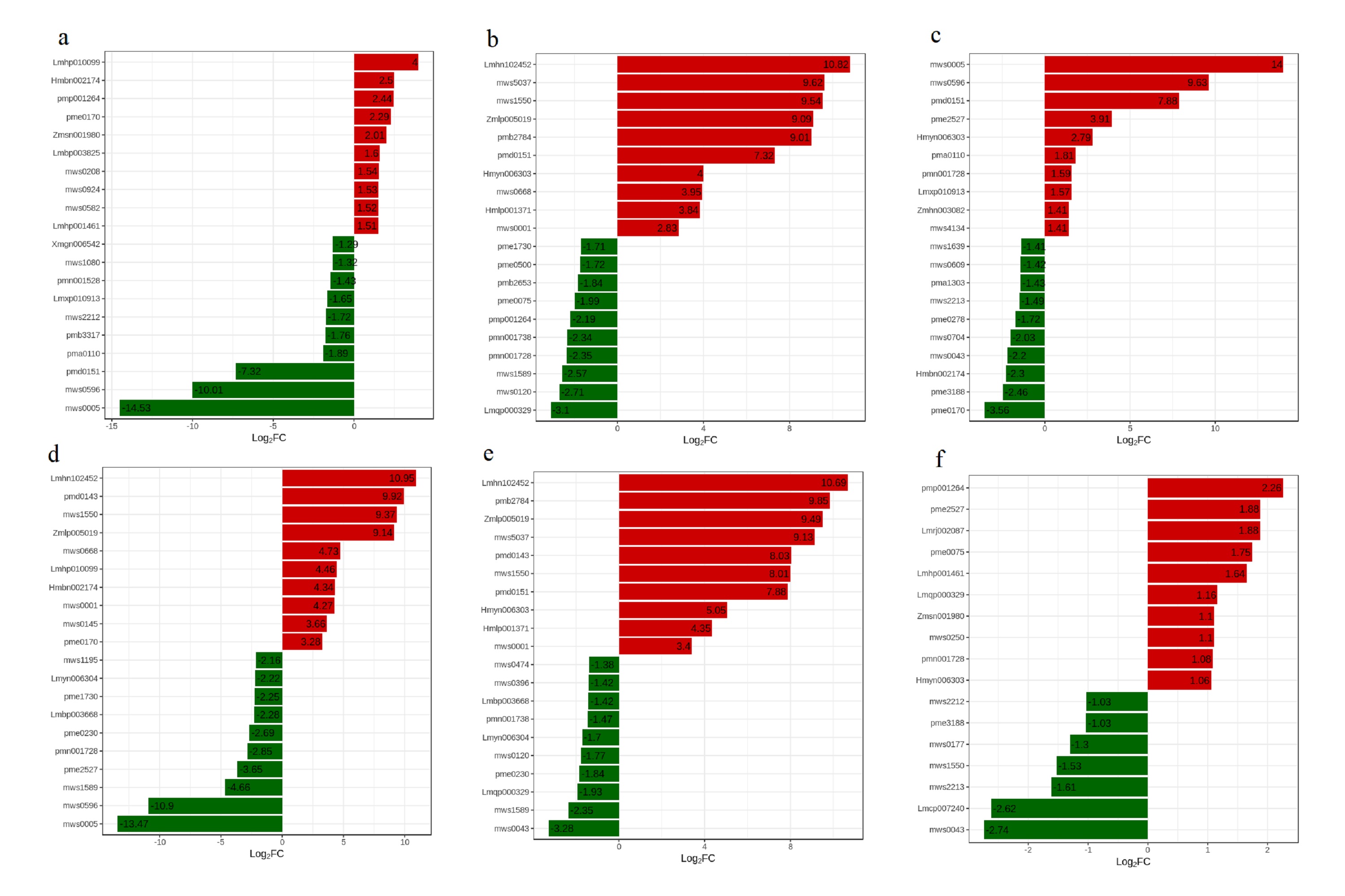

Supplement: Supplementary file 1 [file ijms-22-03294-s001.zip › supplementary files/Figure S3.jpg]
